# The burden of and risk factors for active trachoma in the North and South Wollo Zones of Amhara Region, Ethiopia: a cross-sectional study

**DOI:** 10.1186/s40249-017-0358-3

**Published:** 2017-10-09

**Authors:** Beselam Tadesse, Alemayehu Worku, Abera Kumie, Solomon Abebe Yimer

**Affiliations:** 1 Ethiopian Institute of Water Resources (EIWR), Addis Ababa University, Addis Ababa, Ethiopia; 2 School of Public Health, College of Health Sciences, Addis Ababa University, Addis Ababa, Ethiopia; 3 Department of Microbiology, Oslo University Hospital, Oslo, Norway

**Keywords:** North Wollo, South Wollo, Amhara region, Ethiopia, Trachoma, Prevalence, SAFE strategy, Wash

## Abstract

**Background:**

Trachoma is a disease of the eye, caused by the bacteria *Chlamydia trachomatis*, which can lead to blindness if left untreated. Ethiopia is one of the most trachoma-affected countries in the world. The objective of this study was to determine the prevalence of and associated risk factors for active trachoma among children in selected *woredas* of North and South Wollo Zones in Amhara Region, Ethiopia.

**Methods:**

This study was a community-based, cross-sectional study, which was conducted from October to December 2014 among children aged 1–8. A four-stage random cluster sampling technique was employed to select the study areas and participants. From each selected household, one child was clinically assessed for active trachoma. A structured questionnaire was used to collect sociodemographic, behavioral, and clinical data. Multivariate logistic regression analysis was used to analyze the association between predictor variables and active trachoma.

**Results:**

The overall prevalence of active trachoma among 1358 children was found to be 21.6% (95% *CI*: 19.4–23.8%). When analyzed by the presence or absence of individual WHO simplified system signs of active trachoma, trachomatous inflammation-follicular cases constituted18% (95% *CI*: 15.9–20.2%), while 4.7% (95% *CI*: 3.6–5.8%) were trachomatous inflammation-intense cases. Ocular discharge (a*OR* = 5.2; 95% *CI*: 3.3–8.2), nasal discharge (a*OR* = 1.8; 95% CI: 1.2–2.7), time taken to fetch water (a*OR* = 0.02; 95% *CI*: 0.01–0.05), frequency of hand and face washing (a*OR* = 4.4; 95% *CI*: 1.1–17.8), and access to a latrine (a*OR* = 0.006; 95% *CI*: 0.001–0.030) were found to be independently associated with the presence of active trachoma.

**Conclusions:**

There is a high burden of active trachoma among children in the study areas. Lack of personal hygiene and limited access to a safe water supply and latrines were associated with increased prevalence of active trachoma. In order to reduce the burden of active trachoma, facial cleanliness and environmental improvement components of the SAFE strategy should be upgraded in the study areas.

**Electronic supplementary material:**

The online version of this article (10.1186/s40249-017-0358-3) contains supplementary material, which is available to authorized users.

## Multilingual abstracts

Please see Additional file 1 for translations of the abstract into the five official working languages of the United Nations.

## Background

Trachoma is an eye disease that causes a characteristic roughening of the inner surface of the eyelids and can lead to blindness if left untreated [1]. It is caused by repeated *Chlamydia trachomatis* infections [2]. Trachoma spreads by direct contact with eye and nose discharges from infected individuals or by contact with fomites (inanimate objects that carry infectious agents) such as towels and/or washcloths. Eye-seeking flies can also be a route of mechanical transmission [2].

Untreated, repeated *C. trachomatis* infections result in the development of scar tissue on the inside of the eyelid (the conjunctiva), which pulls the eyelashes inward and rubs against the cornea. This extremely painful condition is known as trichiasis. It is reversible by eyelid surgery, but if left untreated, it could lead to irreversible damage to the eye, corneal opacification, low vision, and blindness [3]. Blinding trachoma is now restricted to areas with poor personal and community hygiene [4].

Several factors are associated with an elevated risk of trachoma. These include lack of water, absence of sanitation facilities, living with a trachoma case, overcrowded living conditions and poverty [1]. Children are the most susceptible groups to infection due to their natural tendency for close contact and face-rubbing behaviors. However, the blinding effects are generally not present until adulthood [5].

Globally, trachoma is responsible for the visual impairment of about 2.2 million people, of whom 1.2 million are irreversibly blind [5]. Ethiopia is one of the most trachoma-affected countries in the world [6]. Of the ten National Regional States in Ethiopia, the Amhara National Regional State (ANRS) is the most severely affected trachoma endemic area of Ethiopia [6]. A study conducted in the North and South Wollo Zones of the ANRS indicated a 52% and 13% overall prevalence, respectively, of trachomatis inflammation-follicular (TF) among children aged 1–9 [7].

The World Health Organization (WHO) recommends the SAFE strategy to control and ultimately eliminate trachoma. This involves: Surgery for trichiasis; Antibiotics for infection; Facial cleanliness to stop transmission; and Environmental improvement, particularly improved access to water and sanitation. The SAFE strategy has been very successful in reducing the number of people with trachoma in many countries, however, trachoma still remains rampant and persistently hyperendemic in the ANRS [8].

Blindness from trachoma results from frequent infections over many years. Ultimate success thus requires the reduction of transmission. The first step to planning an intervention strategy and thus reducing trachoma transmission in a community is to understand the magnitude and associated factors of the disease. Therefore, the objective of the current study was to determine the prevalence and associated factors of active trachoma among children in selected *woredas* of North and South Wollo Zones in the ANRS. This study is part of an ongoing larger study that aims to examine the effect of water, sanitation and hygiene (WASH) interventions on active trachoma elimination in the North and South Wollo Zones of the ANRS, Ethiopia.

## Methods

### Study setting

**Fig. 1:**
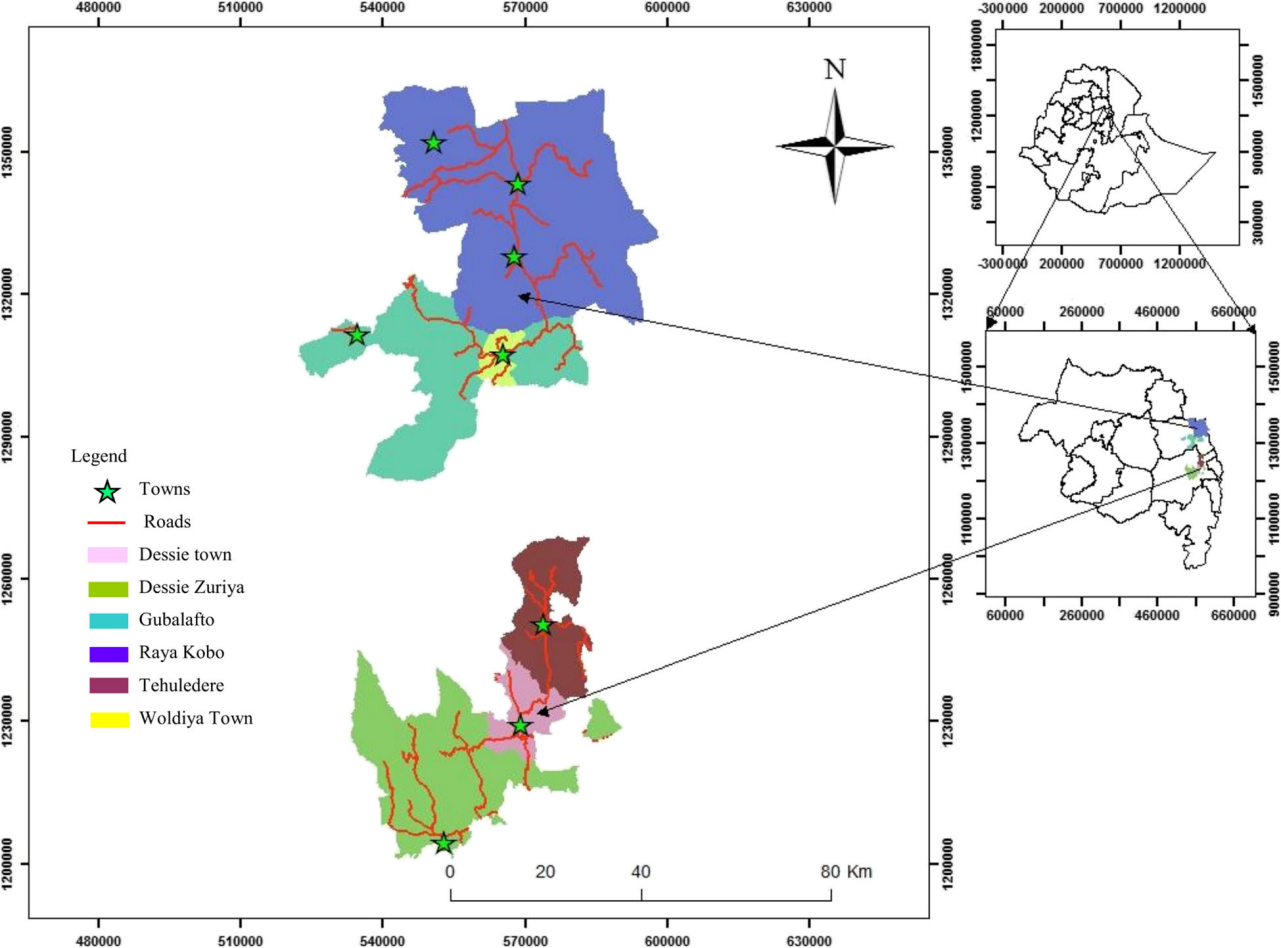
Map showing the ANRS and the study areas

This study was conducted in the North and South Wollo Zones of the ANRS, Ethiopia. Based on the Federal Democratic Republic of Ethiopia Central Statistical Agency [9] population projections, North Wollo Zone has a total estimated population of 1,764,655. The majority (86%) of the population of the zone are rural inhabitants. In terms of religion, 83% of the population practices Orthodox Christianity and the remaining 17% are Muslims [9]. South Wollo Zone has a total estimated population of 2,980,618 [9], with84%of these residing in rural areas. Muslims and Orthodox Christians comprise 71% and 29% of the total population, respectively [9] (see Fig. 1).

The health service coverage in the study areas has reached more than 95% [10]. There are health posts in every *kebele* (smallest administrative unit) designed to serve 5000 people. The health posts are run by two female health extension workers, who are the main grassroots health work forces for health promotion, preventive and basic curative services focused on communicable diseases, and other community-based healthcare interventions.

An increasing number of primary and secondary schools expansion initiatives have been implemented in the study areas in the last decade. In 2014, 76–80% of all eligible students were enrolled in primary schools in North and South Wollo Zones [11]. However, too many children still do not have access to any form of early education programs. The average gross enrolment rate of children aged 4–6 was 41.7% in the region in 2015 [12].

The WHO-recommended WASH program is being implemented in selected *woredas* of the ANRS. A considerable proportion of *woredas* in North and South Wollo Zones are included in this initiative. The main objective of WASH is to increase access to improved water supply and sanitation services for inhabitants in participating *woredas*/towns in the ANRS and Ethiopia at large.

### Study design, sample size, and sampling methods

A community-based, cross-sectional study was conducted among children aged 1–8 in the North and South Wollo Zones of the ANRS, Ethiopia. The current study is part of a larger quasi-experimental study. As children were followed for one year, an age bracket of 1–8 years was considered at baseline.

The sample size was calculated using the EpiInfo™ software package. By considering a 95% confidence interval (*CI*) (Z_α/2_ = 1.96), 5% margin of error, 80% power, design effect of 1.5, 52% prevalence of active trachoma from a previous study [7], a 42% difference in proportion of active trachoma at some future date such that the quantity of p_2_ – p_1_ would be the size of the magnitude of change, and a 10% non-response rate, the total sample size was calculated to be 1358 participants.

A four-stage random cluster sampling technique was employed for selecting the study units. In the first stage, four *woredas* were purposively selected: Raya Kobo and Guba Lafto *woredas* in North Wollo, and Dessie Zuriya and Tehuledere *woredas* in South Wollo. The presence or absence of a WASH program intervention was used as a criterion for selecting and including *woredas* in the study. Accordingly, Raya Kobo and Dessie Zuriya were already selected as intervention *woredas* by the WASH program implementers. Simultaneously, on-WASH intervention *woredas* (Guba Lafto and Tehuledere) were selected as a control group for comparison purposes.

In the second stage, six *kebeles* were randomly selected from each intervention and control *woreda*. The six *kebeles* in the intervention *woredas* were randomly selected out of the 10 intervention *kebeles*. In total, 24 rural *kebeles* were included in the study.

Thirdly, households were randomly selected from each *kebele*. The number of selected households in each *woreda* was decided based on the *woreda*-level population proportion of children.

Fourthly, only one 1–8-year-old child was randomly selected to participate in the study from each randomly selected household. Households where there were no eligible children were excluded from the study.

### Data collection

A semi-structured questionnaire was used to collect the data. Four university graduate data collectors interviewed the study participants. The data collectors were adequately trained regarding the data collection process. The questionnaire was pre-tested in a non-study area and the necessary corrections were made before the actual data collection commenced.

Collected data included sociodemographic, environmental, and behavioral factors associated with active trachoma. Behavioral factors of active trachoma such as face washing were measured by interviewing the guardians of the children diagnosed with active trachoma using a structured questionnaire. Information on variables such as primary source of water, the volume of water consumed per day, and distance to water source was collected from the guardians as well. The volume of water consumed per day was calculated by estimating the volume of fetched water per day and dividing it by the number of family members. A round trip distance to a water source was measured in kilometers or hours. In addition, data on hand and face washing practices and availability of latrine facilities and frequency of latrine use were also collected. A clinical eye examination for the presence of active trachoma was performed for each child participating in the study.

Two nurses who participated as eye examiners in the previously conducted zonal trachoma survey took part in the clinical diagnosis of active trachoma in this study. They were given refresher training on trachoma grading using standard slides showing various grades of trachoma. Standardization of eye examinations for trachoma was then done in community settings. Each nurse was standardized against a highly experienced ophthalmologist in trachoma diagnosis. Each nurse examined 20 children who were rated by a gold standard ophthalmologist. Only those who achieved at least a 60% agreement level with the gold standard were assigned for trachoma grading.

Binocular loupes manufactured by Donegan optical company, inc. at USA (2.5× magnifications) and penlight torches were used during eye examinations. The right eye was examined first, followed by the left eye, in order to avoid failure to recall in which eye the examiner saw an abnormality. Then, the diagnosed children were classified according to the WHO simplified trachoma grading card as either TF, trachomatous inflammation-intense (TI), presence of trachomatous scarring, or free from trachoma. All positive individuals were treated with topical tetracycline eye ointment and were advised to consult the nearby health center for follow-up.

The principal investigator and the supervisors closely monitored the entire data collection process. The filled-out questionnaires and eye examination results were collected after checking for consistency and completeness on a daily basis.

### Operational definitions

**Improved water source** is a water source that, by the nature of its construction or through active intervention, is protected from outside contamination, in particular from contamination with fecal matter.

**Unimproved water source** is a water source that is unprotected from outside contamination, in particular from contamination with fecal matter.

**Latrine** refers to a toilet.

***Woreda*** refers to the third-level administrative entity of Ethiopia.

### Data management and analysis

The raw data were entered using EpiInfo™ version 3.5.1 and exported to the Statistical Package for Social Sciences (SPSS) IBM version 20 (SPSS Inc. Chicago, IL, USA) for analysis. The analysis part involved descriptive statistics (frequency, percentage, mean, and standard deviation, SD); and binary and multivariate analyses. A binary logistic regression analysis was performed to assess the independent effect of each determinant factor after controlling for all other factors. Variables with a *P*-value of less than 0.05 in the bivariate analysis were considered for the multivariate analysis. The magnitude of association between the different variables and presence of active trachoma was measured using odds ratios (*OR*s) with 95% *CI*s of crude odds ratios (c*OR*s) and adjusted odds ratios (a*OR*s).

### Data quality assurance

In this study, several data quality assurance methods were used. Training and field guides were given to the data collectors and supervisors. Pre-tests were made in a non-study area before the actual data collection. Intensive supervision was conducted throughout the survey period by qualified and trained supervisors and the investigator. Questionnaires were checked for consistency and completeness by supervisors at the end of each day.

## Results

### Characteristics of the study participants

In this study, a total of 1358 children aged 1–8 were examined for active trachoma. The mean and SD of their ages was 4.61 ± 2.08 years. Of all children, 53% were females and most of them (75%) were of preschool age, whereas 25% were attending school. Almost all participants were of the Amhara ethnicity. Regarding their religion, 54% and 46% were Orthodox Christians and Muslims, respectively. The overall mean household size was four persons, with household size ranging from one to 12 persons. In terms of the household heads’ education level, 55% were illiterate. Ninety-five percent of the household heads were farmers (see Table 1). The locations of the study sites are presented in Fig. 1.

**Table 1:** Sociodemographic characteristics of the study participants in the intervention and control *woredas* of North and South Wollo Zones of the ANRS, Ethiopia, 2014 (*n* = 1358)

Characteristics	Intervention *woredas* (%)	Control *woredas* (%)	Total (%)
*Primary guardian of child*			
Mother	93.4	96.2	94.8
Grandmother	6.6	3.8	5.2
*Sex of HH head*			
Male	33.5	38.4	35.9
Female	66.5	61.6	64.1
*Marital status of HH head*			
Married	95.6	95.7	95.7
Divorced	3.4	3.5	3.5
Widowed	1	0.7	0.9
*Religion of HH head*			
Orthodox Christian	53.4	55	54.2
Muslim	46.6	45	45.8
*Age of HH head (years)*			
18–29	54.7	47.2	51
30–44	37.8	44.1	40.9
45–59	5.9	7.9	6.9
60 +	1.6	0.7	1.2
*Education status of HH head*			
Illiterate	64.6	44.6	54.6
Literate	35.4	55.4	45.4
*HH head’s primary occupation*			
Farming and/or cattle rearing	95.4	93.8	94.6
Employee	2.7	2.5	2.6
Trade	1.5	3.1	2.3
Daily laborer	0.4	0.6	0.5
*Household size*			
1–3	37.6	33.4	35.5
4–6	50.1	61.3	55.7
≥ 7	12.2	5.3	8.8
*Child’s age*			
1–3	32.9	35	33.9
4–6	41.4	43.2	42.3
7–8	25.7	21.8	23.7
*School grade of child*			
0	77.7	71.5	74.6
≥ 1	22.3	28.5	25.4
*Sex of child*			
Male	46.2	47.4	46.8
Female	53.8	52.6	53.2

*HH* household

### WASH status of the study participants

Sixty-eight percent of children were from families that had improved water sources and the time it took to fetch water was ≤30 min of walking for 51% of the households. Daily water consumption per person for 57% of the households was found to be not greater than 10 l. Fifty-seven percent of children were from households that did not have access a latrine (see Table 2).

**Table 2:** WASH status of study participants in the intervention and control *woredas* of North and South Wollo Zones of the ANRS, Ethiopia, 2014 (*n* = 1358)

Characteristics	Intervention *woredas* (%)	Control *woredas* (%)	Total (%)
*Source of water*			
Improved	77.4	59.3	68.3
Unimproved	22.6	40.7	31.7
*Time taken to fetch water*			
≤ 30 min	63.6	39	51.3
> 30 min	36.4	61	48.7
*Water consumption/person/day (in liters)*			
≤ 10	56.3	58.4	57.4
11–20	40.1	41.5	40.8
> 20	3.5	0.1	1.8
*Latrine access*			
Yes	46.3	39	42.6
No	53.7	61	57.4
*Hand washing container near latrine*			
Yes	3.8	4.4	4.1
No	96.2	95.6	95.9

### Hand and face washing practices of the study participants

The main reason for hand and face washing for half of the respondents was to have good health, whereas for the remaining 50% it was to maintain the beauty of their face and hands. The majority (91%) of the guardian of a child reported that they washed theirs and their children’s hands and faces once a day. Eighty percent of the guardian of a child did not use soap to wash their hands and face (see Table 3).

**Table 3:** Hand and face washing practices of the study participants in the intervention and control *woredas* of North and South Wollo Zones of the ANRS, Ethiopia, 2014 (*n* = 1358)

Characteristics	Intervention *woredas* (%)	Control *woredas* (%)	Total (%)
*Reasons of washing hands and face*			
Beauty	54.7	45.1	49.9
Good health	45.3	54.9	50.1
*Frequency of washing hands and face*			
Once	94.2	88.1	91.2
≥ Two times a day	5.8	11.9	8.8
*Soap use during hand and face washing*			
Yes	28.9	10.4	19.7
No	71.1	89.6	80.3

### Prevalence of active trachoma

**Fig. 2:**
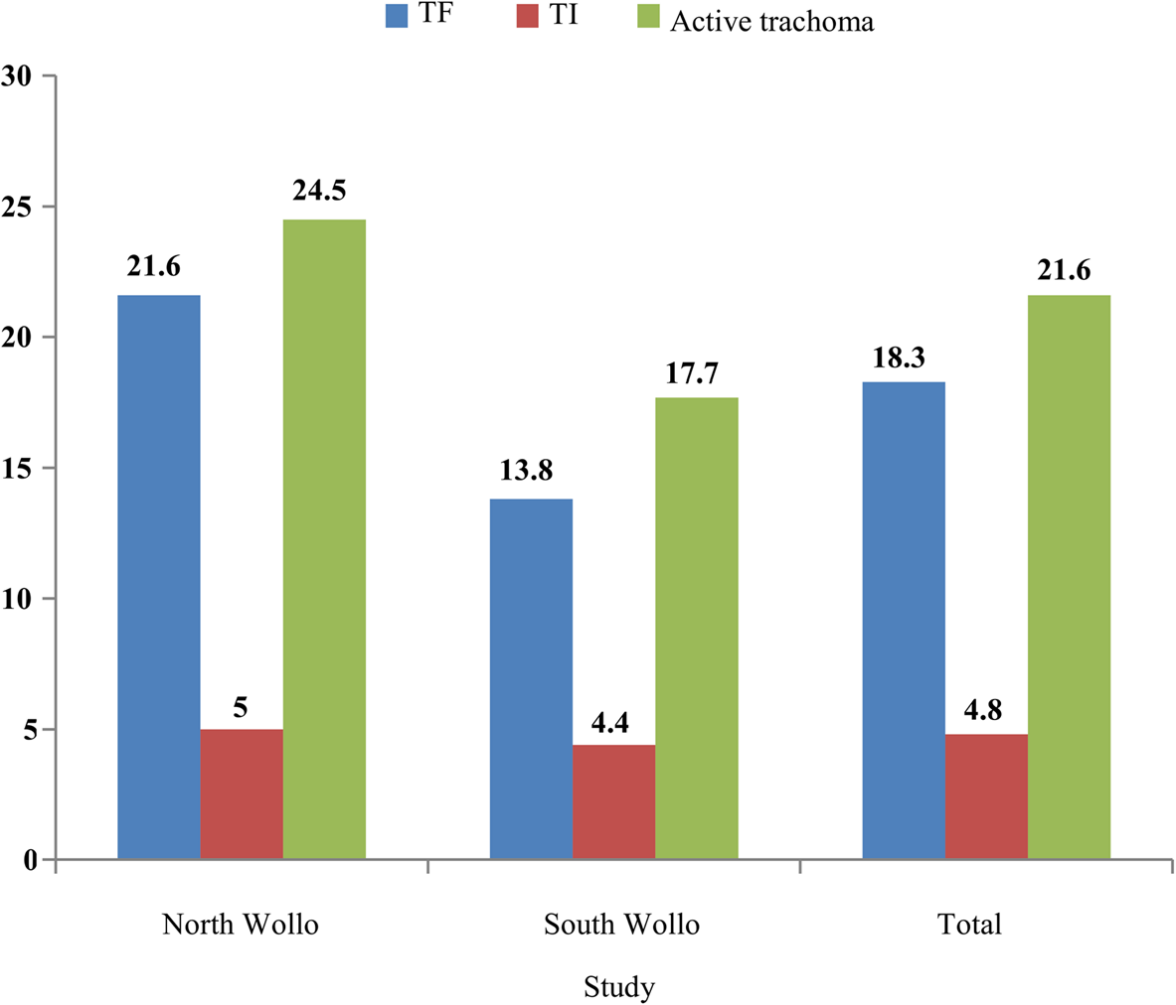
Prevalence of active trachoma among children aged 1–8 in the North and South Wollo Zones of the ANRS, Ethiopia, 2014

The overall prevalence of active trachoma among children aged 1–8 was 21.6% (95% *CI*: 19.4–23.8%). There were 18% (95% *CI*: 15.9–20.2%) of TF cases, while 4.7% (95% *CI*: 3.6–5.8%) were TI cases (see Fig. 2).

**Fig. 3:**
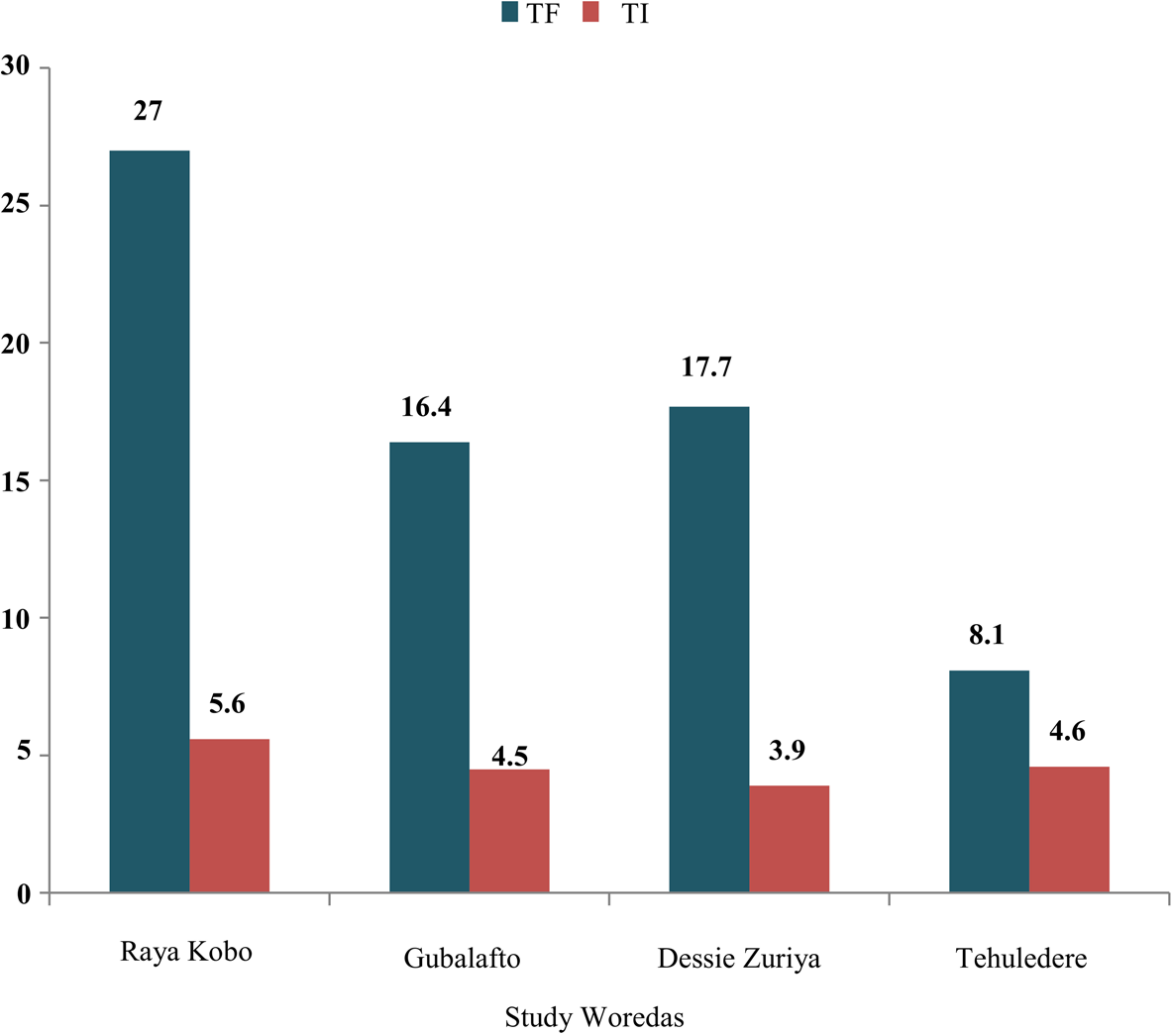
*Woreda*-level prevalence of active trachoma among children aged 1–8 in the North and South Wollo Zones of the ANRS, Ethiopia, 2014

**Fig. 4:**
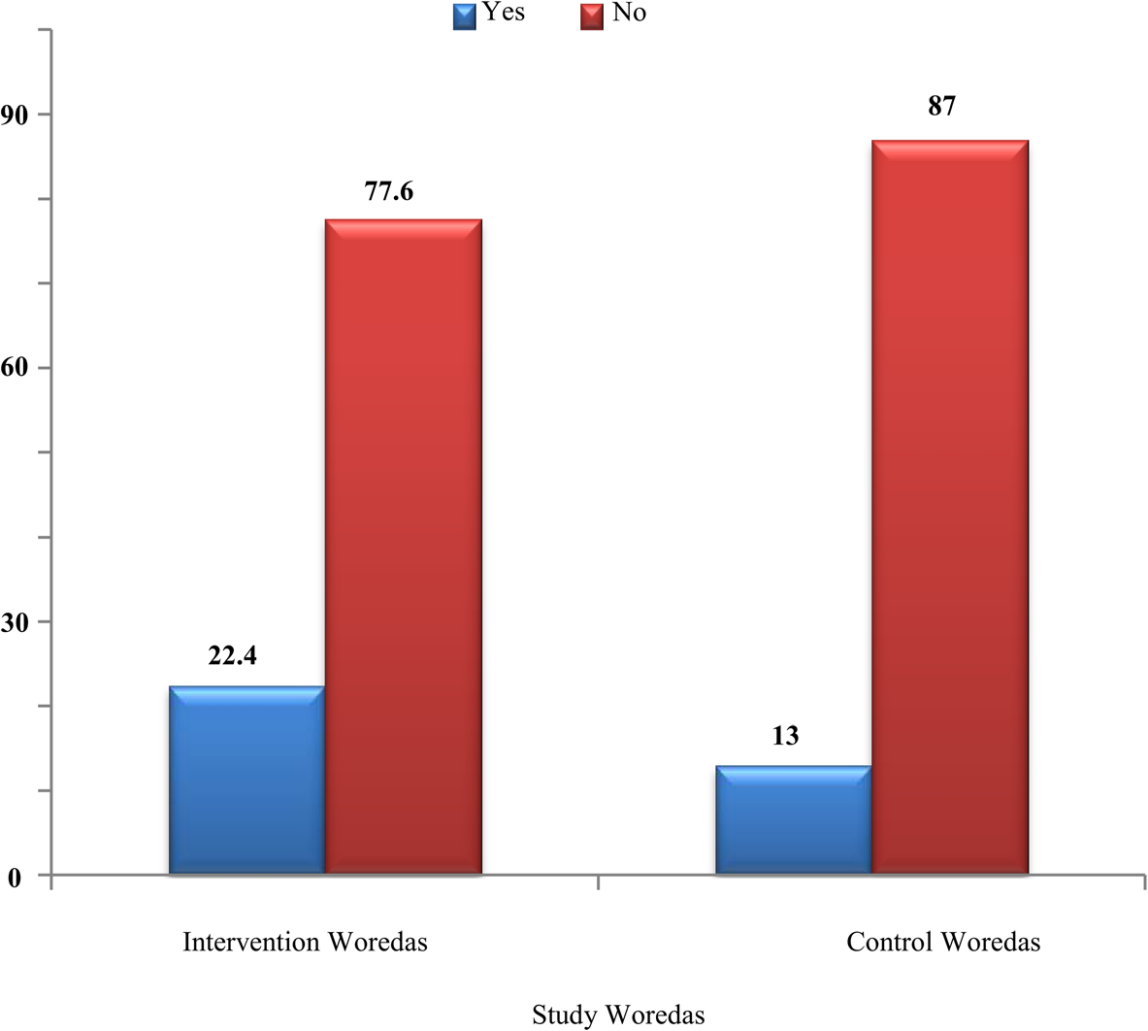
Proportions of children aged 1–8 with active trachoma in the intervention and control *woredas* of the North and South Wollo Zones of the ANRS, Ethiopia, 2014

The prevalence of TF ranged from 8.1% to 27% in the study *woredas*. The highest prevalence (27%) was found in Raya Kobo. The second highest (17.7%) was found in Dessie Zuriya, followed by 16.4% and 8.1% prevalence rates in Guba Lafto and Tehuledere, respectively (see Fig. 3).In the intervention and control *woredas*, 22.3% and 13% of the populations, respectively, had TF cases (see Fig. 4).

### Factors associated with the prevalence of active trachoma

The odds of having active trachoma among children who had ocular discharge were five times higher than those who did not have this (a*OR* = 5.2; 95% *CI*: 3.3–8.2). In addition, nasal discharge (a*OR* = 1.8; 95% *CI*: 1.2–2.7), time to fetch water (aOR = 0.02; 95% *CI*: 0.01–0.05), frequency of hand and face washing (a*OR* = 4.4; 95% *CI*: 1.1–17.8), and access to latrine (a*OR* = 0.006; 95% *CI*: 0.001–0.03) were independently associated with the presence of active trachoma (see Tables 4 and 5).

**Table 4:** Bivariate analysis showing the association between sociodemographic and environmental factors and active trachoma in the North and South Wollo Zones of the ANRS, Ethiopia, 2014 (*n* = 1358)

Variables	Active trachoma, number		c*OR* (95% *CI*)
	Yes	No	
*Sex of child*			
Female	153	568	0.948 (0.732–1.227)
Male	141	496	1
*Child’s age*			
1–3	108	350	1.24 (0.879–1.759)
4–6	121	452	1.079 (0.770–1.512)
7–8	65	262	1
*Ocular discharge*			
Yes	124	111	6.262 (4.62–8.48) *
No	170	953	1
*Nasal discharge*			
Yes	127	239	2.625 (2.0–3.45) *
No	167	825	1
*Primary guardian of child*			
Grandmother	28	43	2.499 (1.524–4.099) *
Mother	266	1021	1
*Education status of HH head*			
Literate	131	485	0.959 (0.74–1.24)
Illiterate	163	579	1
*Religion of HH head*			
Orthodox Christian	175	560	1.324 (1.02–1.72)
Muslim	119	504	1
*Household size*			
1–3	105	377	0.826 (0.52–1.32)
4–6	159	598	0.789 (0.503–1.24)
≥ 7	30	89	1
*HH primary water source*			
Improved	201	726	1 (0.76–1.33)
Unimproved	93	338	1
*Time to fetch water*			
≤ 30 min	7	515	0.026 (0.012–0.056)
> 30 min	287	549	1
*Frequency of hand and face washing*			
Once a day	291	990	7.25 (2.27–23.17) *
Two or more times a day	3	74	1
*Access to latrine*			
Yes	2	576	0.006 (0.001–0.023) *
No	292	488	1
*Frequency of latrine usage*			
Sometimes	40	129	1.325 (0.877–2.003)
Always	106	453	1

*HH* household

**Table 5:** Multiple covariate analysis showing the association between sociodemographic and environmental factors and active trachoma in the North and South Wollo Zones of the ANRS, Ethiopia, 2014 (*n* = 1358)

Variables	Active trachoma (*N*)		a*OR* (95% *CI*)
	Yes	No	
*Ocular discharge*			
Yes	124	111	5.18 (3.27–8.21)
No	170	953	1
*Nasal discharge*			
Yes	127	239	1.79 (1.21–2.66)
No	167	825	1
*Primary guardian of child*			
Grandmother	28	43	2.35 (1.09–5.06)
Mother	266	1021	1
*Time to fetch water*			
≤ 30 min	7	515	0.02 (0.01–0.05)
> 30 min	287	549	1
*Frequency of hand and face washing*			
Once a day	291	990	4.44 (1.1–17.77)
≥ Two times a day	3	74	1
*Access to latrine*			
Yes	2	576	0.006 (0.001–0.03)
No	292	488	1

## Discussion

The overall prevalence of active trachoma among children aged 1–8 in the current study was found to be 21.6%, which is higher than the WHO trachoma elimination target (a prevalence of active trachoma (grade TF) in children aged 1–9 years of <5%) [13]. The WHO endorsed an integrated SAFE strategy to combat the transmission of trachoma. However, even if this important strategy has been implemented for more than 10 years in trachoma hyperendemic areas of North and South Wollo Zones, the current study shows that the prevalence of active trachoma has not reached the elimination target. This may be related to the weak integration of health promotion activities with personal and environmental hygiene and sanitation practices.

The burden of active trachoma observed in the current study was almost in agreement with other Ethiopian cross-sectional studies that showed prevalence rates of 24.1% and 24.5% in the Baso Liben District of East Gojjam Zonefrom February to April 2012 [14] and Jimma Zone from November 1994 to January 1995 [15], respectively. Our finding is also a little lower than the prevalence rate of 25.1% reported in Malawi [16]. On the other hand, the prevalence of active trachoma found in this study was significantly lower than what was found in some studies conducted in other parts of Ethiopia. For example, in 2001, 40.9% of active trachoma prevalence among children was reported in the South Wollo Zone [17]. Another cross-sectional study conducted in a highland population of Gondar from October to December 1998 revealed a 59.0% prevalence of active trachoma [18]. Bejiga and Alemayehu reported a prevalence of 51.5% in 2001 in Dalocha District, Central Ethiopia [19]. The relatively lower prevalence of active trachoma in our study might be related to the success of the SAFE strategy in the ANRS, the water resource development activities provided by national and regional programs, and the health education activities provided by other stakeholders.

In contrast, the burden of active trachoma in our study was higher than the 12% and 15.6% prevalence rates reported in the Dangla and Dera *woredas* of the west and northern part of the ANRS that were conducted from March to April 2014, respectively [20, 21]. This might be related to good infrastructure and better availability of water in Dangla. In Dera, the SAFE strategy has been implemented for the last 10 years and this may have resulted in a significant reduction of active trachoma prevalence.

The WHO recommends a reduction of TF cases to less than 5% among children aged 1–9to eliminate blinding trachoma. However, the number of active trachoma cases in the study districts of Raya Kobo, Guba Lafto, and Dessie Zuriya exceeded 10%, while it was 8.1% in Tehuledere. This finding calls for an urgent need for mass distribution campaigns of tetracycline eye ointment or azithromycin oral antibiotics to reduce the transmission of trachoma in these *woredas*.

A number of predictor variables were significantly associated with the prevalence of active trachoma in this study. A significant association was observed between active trachoma and occurrence of ocular and nasal discharge. This finding is similar to the baseline results of cluster randomized controlled trial studies conducted between May and November 2008. These studies identified the presence of ocular and nasal discharge as risk factors for the presence of fly on eyes and active trachoma in Tanzania and Ethiopia [22, 23]. Unlike the findings from the southern part of Ethiopia, no association was observed between the educational status of children and occurrence of active trachoma [24].

Family size was not associated with the occurrence of active trachoma in this study. The risk of acquiring trachoma is related to the likelihood of contact with an infected individual rather than being a member of a large family [25].

Children from families that spend more than 30 min walking to a water source were more likely to have active trachoma than those that spend less than 30 min. This finding is in line with results from cross-sectional studies conducted in Ethiopia and Tanzania [26–28]. Similar findings have also been documented in China, India, and Malawi [29–31].

Water sources (measured as improved/unimproved) did not appear to be a determinant factor for the presence of active trachoma in this study. Since trachoma is a water-washed disease, the availability of water seems to be more critical than the type of water source in reducing trachoma.

Contrary to the studies conducted in Ankober in July 2007 and Baso Liben in 2012 [14, 28], this study revealed higher associations between the presence of active trachoma and the absence of a latrine. This could be associated with the presence of open-field feces as a breeding media for the trachoma fly vector *Musca sorbens* that leads to a higher chance of transmission.

The major limitation of our study is that the estimations of household fetched water volume per day and time taken to fetch water were merely based on the respondents’ response to the interviewer questions, which may be uncertain. We attempted to minimize the errors due to this by asking different individuals from households to answer the same question and taking the average value into account.

## Conclusions

The WHO endorsed an integrated SAFE strategy to control and ultimately eliminate trachoma. The four components of the SAFE strategy must be implemented with equal attention for successful trachoma control. However, even though this important strategy has been implemented for more than 10 years in the trachoma hyperendemic areas of North and South Wollo Zones, the prevalence of active trachoma has not reached the WHO elimination target.

This study showed that trachoma is still hyperendemic in the North and South Wollo Zones of the ANRS. A number of risk factors including ocular discharge, nasal discharge, longer time to fetch water and access to a latrine were significantly associated with active trachoma. Trachoma can be controlled and eliminated when communities experience better health-seeking behavior, and personal hygiene and sanitary conditions. Therefore, in order to reduce the burden of active trachoma in the study areas, all components of the SAFE strategy must continue to be efficiently implemented and even scaled-up.

## Additional file

Additional file 1: Multilingual abstracts in the five official working languages of the United Nations. (PDF 768 kb)

## Abbreviations

ANRS: Amhara National Regional State
AOR: adjusted odds ratio
*CI*: confidence interval
c*OR*: crude odds ratio
*OR*: odds ratio
*SD*: standard deviation
SPSS: Statistical Package for Social Sciences
TF: trachomatous inflammation-follicular
TI: trachomatous inflammation-intense
WASH: water, sanitation and hygiene
WHO: World Health Organization
