# Supplementary material for: The burden of and risk factors for active trachoma in the North and South Wollo Zones of Amhara Region, Ethiopia: a cross-sectional study
Source: Infect Dis Poverty. 2017 Oct 9;6:143. doi: 10.1186/s40249-017-0358-3 (PMC5632837; doi:10.1186/s40249-017-0358-3)
Supplement: Additional file 1: — Multilingual abstracts in the five official working languages of the United Nations. (PDF 768 kb) [file 40249_2017_358_MOESM1_ESM.pdf]

Translation of the abstract into the five official working languages of the United Nations

عبء وعوامل الخطر المتعلقة بمرض الرمد الحبيبي النشط في شمال وجنوب مناطق ووالو في أقليم أمهره في أثيوبيا: دراسة شاملة

بيسيلام تاديسي، واليمايهو وركو، وكامي أبيرا، وسولومون أبيبي ييمر

ملخص
لمحة عامة: <bx/> الرمد الحبيبي هو مرض للعين تسببه البكتيريا *المتدثرة الحثرية، والذي يمكن أن يؤدي إلى العمى إذا ترك دون علاج. وأثيوبيا هي واحدة من أكثر البلدان تضررًا من الرمد الحبيبي في العالم. وكان الهدف من هذه الدراسة هو تحديد مدى انتشار الرمد الحبيبي النشط وعوامل الخطر المرتبطة به بين الأطفال في المقاطعات المختارة من شمال وجنوب مناطق واولو في أقليم أمهرة في أثيوبيا .*
الأساليب: كانت هذه الدراسة دراسة مجتمعية شاملة، والتي أجريت من أكتوبر إلى ديسمبر 2014 بين الأطفال الذين تتراوح أعمارهم بين 1-8. تم استخدام تقنية أخذ العينات العشوائية من أربع مراحل لتحديد مناطق الدراسة والمشاركين. من كل أسرة مختارة، تم تقييم طفل سريريًا بالرمد الحبيبي النشط. واستخدم استبيان منظم لجمع البيانات الاجتماعية الديموغرافية والسلوكية والسريرية. تم استخدام تحليل الانحدار اللوجستي متعدد المتغيرات لتحليل الارتباط بين متغيرات التنبؤ والرمد الحبيبي النشط.
النتائج: وقد وجد أن الانتشار الإجمالي للرمد الحبيبي بين 1 358 طفلًا هو 21.6٪ (95٪ 23.8٪ - 19.4 :*CI*). عندما تمالتحليل فيوجود أو عدم وجود نظام مبسط فردي تابع لمنظمة الصحة العالمية وُجدت عوارض للرمد الحبيبي النشط، وحالات التهاب الجريبات التراخومية تشكل 18٪ (95٪ 20.2٪ - 15.9 :*CI*)، في حين أن 4.7٪ (95٪ 5.8٪ - 3.6 :*CI*) كانت حالات حادة الالتهاب. . تم ربط الإفرزات العينية (*أو* = 5.2؛ 95٪ 8.2 - 3.3 :*CI*)، الإفرازات الأنفية(*أو*= 1.8. 95٪ سي: 1.2-2.7)، والوقت المستغرق في جلب الماء (*أو* = 0.02؛ 95٪ 0.05 - 0.01 :*CI*)، معدل غسل اليد والوجه (*أو* = 4.4؛ 95٪ 1.1 :*CI* - 17.8)، والوصول إلى المرحاض (*أو*= 0.006. 95٪ 0.03 - 0.001 :*CI*) ربطًا مستقلًا بوجود رمد حُبيبي نشط.
الاستنتاجات: هناك عبء عالي بخصوص رمد حبيبي نشط بين الأطفال في مناطق الدراسة. ارتبط نقص النظافة الشخصية والوصول المحدود إلى إمدادات المياه الصالحة للشربوالمراحيض بزيادة انتشار الرمد الحبيبي النشط. من أجل الحد من عبء الرمد الحبيبي النشط، يجب تحسين نظافة الوجه ومكونات تحسين البيئة التابعة لاستراتيجية SAFE في مناطق الدراسة.

Translated from English version into Arabic by Eman Shahen, through

TRANSLATORS
WITHOUT BORDERS

埃塞俄比亚阿姆哈拉地区沃罗区域的北部和南部地区活动性沙眼的疾病负担和风险因素：一项横断面研究

Beselam Tadesse, Alemayehu Worku, Abera Kumie, Solomon Abebe Yimer

摘要
引言：沙眼是由沙眼衣原体引起的眼病，如果不及时治疗会导致失明。埃塞俄比亚是世界上沙眼感染最严重的国家之一。本研究的目的在于确定埃塞俄比亚阿姆哈拉地区沃罗区域的北部和南部所选地区儿童活动性沙眼的患病率及相关危险因素。
方法：本研究是于 2014 年 10 月到 12 月，在 1–8 岁儿童中进行的一项基于社区的横断面研究。采用四阶段随机整群抽样的方法选择研究区域和参与者。每个选定家庭中的一个孩子进行活动性沙眼的临床评估。使用结构化问卷收集社会人口学、行为学和临床数据。采用多元 logistic 回归分析预测变量与活动性沙眼的相关性。
结果：在 1 358 名儿童中，活动性沙眼的总患病率为 21.6%(95% *CI*: 19.4–23.8%)。使用 WHO 沙眼简化分级系统来分析活动性沙眼的症状，沙眼性炎症-滤泡病例占 18 %（95% *CI*：15.9–20.2%），而重度沙眼性炎症占 4.7%（95% *CI*：3.6–5.8%）。眼分泌物（a*OR* = 5.2；95% *CI*：

3.3–8.2）、鼻分泌物（a*OR* = 1.8；95% *CI*：1.2–2.7）、取水时间（a*OR* = 0.02；95% *CI*：0.01–0.05）、洗手和洗脸的频率（a*OR* = 4.4；95% *CI*：1.1–17.8）以及使用公共厕所（a*OR* = 0.006；95% *CI*：0.001–0.03）与活动性沙眼独立相关。
结论：该研究区域儿童的沙眼疾病负担很重。个人卫生状况不佳、安全饮用水和公共厕所的缺乏与活动性沙眼患病率升高有相关性。为了减轻活动性沙眼的疾病负担，面部清洁和改善环境作为 SAFE 策略的组成部分，应在研究区域内着重开展这两方面的工作。

Translated from English version into Chinese by Zhou Guan, edited by Pin Yang

CHINESE CENTER FOR DISEASE CONTROL AND PREVENTION
NATIONAL INSTITUTE OF PARASITIC DISEASES

Charge de morbidité et facteurs de risque du trachome actif dans les zones Wollo Nord et Wollo Sud de la région d'Amhara (Éthiopie) : étude transversale

Mouhssine Tadesse, Alemayehu Worku, Abera Kumie Solomon Abebe Yimer

Résumé

Informations générales : le trachome est une affection de l'œil, causée par la bactérie *Chlamydia trachomatis*, qui peut entraîner la cécité si elle n'est pas traitée. L'Éthiopie est un des pays les plus touchés du monde par le trachome. La présente étude avait pour objet de déterminer la prévalence du trachome actif et les facteurs de risque associés parmi les enfants de certaines *woredas* des zones Wollo Nord et Wollo Sud de la région d'Amhara (Éthiopie).
Méthodes : cette étude transversale axée sur les communautés a été menée d'octobre à décembre 2014 chez des enfants âgés de 1 à 8 ans. Les zones et les participants à étudier ont été sélectionnés par échantillonnage aléatoire à grappes à quatre degrés. Dans chacun des ménages sélectionnés, un enfant a été cliniquement évalué pour le trachome. Un questionnaire structuré a servi à collecter des données sociodémographiques, comportementales et cliniques. Les associations entre variables prédictives et trachome ont été étudiées par analyse de régression logistique multivariée.
Résultats : on a constaté une prévalence globale du trachome actif parmi 1 358 enfants de 21,6% (*IC* à 95% : 19,4 – 23,8%). Analysés en fonction de la présence ou de l'absence des signes individuels de trachome actif retenus dans le système simplifié de codage de l'OMS, les cas d'infection trachomateuse folliculaire constituaient 18% (*IC* à 95% : 15,9 – 20,2%), et 4,7% (*IC*à 95% : 3,6 – 5,8%) étaient des cas d'inflammation trachomateuse intense. On a constaté que les facteurs ci-après étaient en association indépendante avec le trachome actif : écoulement oculaire (quotient de probabilité ajusté (A*OR*] = 5,2 ;*IC*à 95 % : 3,3 – 8,2), écoulement nasal (*quotient de probabilité ajusté*= 1,8 ; IC à 95% : 1,2 – 2,7), temps nécessaire pour chercher de l'eau (*quotient de probabilité ajusté* = 0,02 ;*IC* à 95 % : 0,01 – 0,05), fréquence du lavage des mains et du visage (*quotient de probabilité ajusté* = 4.4 ; *IC* à 95 % : 1,1 – 17,8) accès à une latrine (*quotient de probabilité ajusté*= 0,006 ; *IC* à 95 % : 0,001 – 0,03).
Conclusions : parmi les enfants des zones étudiées, la charge de morbidité du trachome actif est élevée. Le défaut d'hygiène personnelle et l'accès limité à l'approvisionnement en eau salubre et aux latrines ont été associés à une augmentation de la prévalence du trachome actif. Pour réduire la charge de morbidité du trachome actif, il faudrait développer les composantes Nettoyage du visage et Changement de l'environnement de la stratégie CHANCE dans les zones étudiées.

Translated from English version into French by Sinany, through

Бремя и факторы риска активной трахомы в Северном и Южном Уолло региона Амхара, Эфиопия: перекрёстное исследование

Беселам Тадессе, Алемайеху Уорку, Абера Куми, Соломон Абебе Йимер

Аннотация

Справочная информация: Трахома - это заболевание глаз, вызываемое бактерией *Chlamydia trachomatis*и при отсутствии лечения способное привести к слепоте. Эфиопия является одной из наиболее пострадавших от трахомы стран в мире. Цель этого исследования состояла в том, чтобы определить распространенность активной трахомы среди детей в отдельных *округах* Северного и Южного Уолло Амхары в Эфиопии, а также связанные с ней факторы риска.

Методы: Данный анализ представляет собой перекрестное исследование, проводимое на уровне общин с октября по декабрь 2014 года среди детей в возрасте от 1 до 8 лет. Для отбора участников и областей исследования была применена четырехэтапная методика случайного кластерного отбора. Один ребенок из каждой отобранной семьи был клинически диагностирован на присутствие активной трахомы. Для сбора социодемографических, поведенческих и клинических данных использовалась структурированная анкета. С целью изучения взаимосвязи между переменными прогноза и активной трахомой был задействован многомерный анализ логической регрессии.

Результаты: Общая распространённость активной трахомы среди 1 358 детей составила 21,6% (95% *CI*: 19,4 – 23,8%). При анализе на наличие или отсутствие симптомов активной трахомы по отдельной упрощенной системе ВОЗ, случаи трахоматозного фолликулярного воспаления составили 18% (95% *CI*: 15,9 – 20,2%), в то время как 4,7% (95% *CI*: 3,6 – 5,8%) составили случаи острого трахоматозного воспаления. Выделения из глаза (a*OR* = 5,2; 95% *CI*: 3,3 – 8,2), из носа (*OR*= 1,8; 95% Cl: 1,2-2,7), время, необходимое для извлечения воды (a*OR* = 0,02; 95% *CI*: 0,01 – 0,05), частота мытья рук и лица, (a*OR* = 4,4; 95% *CI*: 1,1 – 17,8) а также доступ к уборной (a*OR*= 0,006; 95% *CI*: 0,001 – 0,03) оказались во взаимосвязи с присутствием активной трахомы.

Выводы: У детей в исследуемых районах был обнаружен высокий уровень бремени активной трахомы. Отсутствие личной гигиены, а также ограниченный доступ к безопасным источникам водоснабжения и уборным оказались напрямую связанными с распространяемостью активной трахомы. С целью снижения бремени активной трахомы в изучаемых районах необходимо привести гигиену лица и улучшение компонентов окружающей среды в соответствие с разработанной ВОЗ стратегией SAFE.

Translated from English version into Russian by Liudmila Tomanek, through

Prevalencia y factores de riesgo del tracoma activo en las zonas Wollo del Norte y del Sur de la región de Amhara, Etiopía: un estudio transversal.

Beselam Tadesse, Alemayehu Worku, Abera Kumie, Salomón Abebe Yimer

Resumen

Antecedentes: el tracoma es una enfermedad ocular, provocada por la bacteria *Chlamydia trachomatis* que, si no se trata, puede producir ceguera. Etiopía es uno de los países más afectados por el tracoma del mundo. El objetivo de este estudio fue determinar la prevalencia y los factores de riesgo asociados con el tracoma activo entre los niños de los *woredas (distritos)* seleccionados en las zonas de Wollo del Norte y del Sur de la región de Amhara, Etiopía.

Metodología: se trató de un estudio transversal, basado en la comunidad, que se llevó a cabo entre octubre y diciembre de 2014 entre niños de 1 a 8 años. Se utilizó una técnica de muestreo por conglomerados aleatoria de cuatro etapas para seleccionar las áreas de estudio y los participantes. En cada hogar seleccionado, se llevó a cabo la evaluación clínica de un niño para detectar tracoma activo. Se empleó un cuestionario estructurado para recopilar datos sociodemográficos, conductuales y clínicos. Se utilizó un análisis de regresión logística multivariable para analizar la relación entre las variables predictivas y el tracoma activo.

Resultados: la prevalencia global de tracoma activo entre 1 358 niños fue del 21,6% (*IC* del 95%: 19,4 - 23,8%). Cuando se llevó a cabo el análisis para detectar la presencia o ausencia de signos individuales según el sistema de clasificación simplificado de tracoma activo de la OMS, se observó que el 18% de los casos fueron de inflamación tracomatosa folicular (*IC* del 95%: 15,9 - 20,2%), mientras que el 4,7% (*IC* del 95%: 3,6 - 5,8%) fueron casos de inflamación tracomatosa intensa. Se descubrió que la secreción ocular (a*OR* = 5,2; *IC* del 95%: 3,3 - 8,2), la secreción nasal (a*OR*= 1,8; IC del 95%: 1,2-2,7), el tiempo empleado para recoger agua (a*OR* = 0,02; *IC* del 95%: 0,01 - 0,05), la frecuencia de lavado de manos y cara (a*OR* = 4,4; *IC* del 95%: 1,1 - 17,8), y el acceso a una letrina (a*OR*= 0,006; *IC* del 95%: 0,001 - 0,03) se relacionan de manera independiente con la presencia de tracoma activo.

Conclusiones: existe una carga elevada de tracoma activo entre los niños de las áreas de estudio. Se relacionó la falta de higiene personal y el acceso limitado a un suministro de agua potable y letrinas con una mayor prevalencia de tracoma activo. Para reducir la carga de tracoma activo, es necesario mejorar los hábitos de limpieza facial y los componentes para producir mejoras ambientales de la estrategia SAFE, en las áreas donde se llevó a cabo el estudio.

Translated from English version into Spanish by Maria Paula Gorgone, through

TRANSLATORS
WITHOUT BORDERS
